# Impact of diurnal temperature variations on sputum bacterial detection in hospitalized patients with acute COPD exacerbation: a retrospective study from Fuzhou, China

**DOI:** 10.1186/s12890-024-03102-w

**Published:** 2024-06-22

**Authors:** Hong Xue, Qing Xue, Chunhui Wang, Qianshun Chen, Daxuan Wang, Zhen Li, Baosong Xie, Wei Zheng

**Affiliations:** 1 Department of Respiratory and Critical Care Medicine, Provincial School of Clinical Medicine, Fujian Medical University, Fujian Provincial Hospital, Fuzhou University Affiliated Provincial Hospital, No. 134 East Street, Gulou District, Fuzhou City, 350001 Fujian Province China; 2 The Third Clinical Medical College, Fujian Medical University, Ningde Municipal Hospital, Ningde, 350021 Fujian China; 3 Fujian Meteorological Service Centre, Fujian Meteorological Bureau, Fuzhou City, 350001 Fujian Province China; 4 Department of Thoracic Surgery, Provincial School of Clinical Medicine, Fujian Provincial Hospital, Fujian Medical University, Fuzhou City, 350001 Fujian Province China; 5 Microbiology Laboratory, Provincial School of Clinical Medicine, Fujian Provincial Hospital, Fujian Medical University, Fuzhou City, 350001 Fujian Province China

**Keywords:** Chronic obstructive pulmonary disease, Acute exacerbation, Diurnal temperature differences, Sputum pathogens culture, Respiratory infections

## Abstract

**Objective:**

To investigate the association between meteorological data three days before admission and the status of sputum pathogens culture in hospitalized patients with Acute exacerbation of Chronic obstructive pulmonary disease (AECOPD) and respiratory infections.

**Methods:**

Data from 1,370 AECOPD patients (80.66% males, approximately 80% age > 70) with respiratory infections hospitalized in Fujian Provincial Hospital between December 2013 and December 2019 were collected. This cohort comprised, along with concurrent meteorological data from Fuzhou. Group differences were analyzed to compare the meteorological data three days prior to admission between patients with positive sputum pathogen cultures and those without. Logistic regression models were employed to investigate the association between meteorological parameters and the status of sputum pathogen cultures in patients with AECOPD and respiratory infections. Sensitivity analyses was conducted among the hospitalized patients from 2013 to 2016 and 2017–2019. Stratified analysis was performed to explore the factors affecting the effect of temperature differences and their interactions.

**Results:**

578(42.19%) cases had a positive sputum culture report indicating pathogen growth. 323 cases were found with Gram-negative bacteria, 160 with Gram-positive bacteria, and 114 with fungi. Uni-variate analysis revealed statistical differences in DTD three days prior to admission (DTD-3d) between the positive and negative sputum culture groups (*p* = 0.019). Multivariate analysis indicated that an increase in the risk of positive sputum pathogen cultures was associated with greater DTD three days before admission (DTD-3d), with OR1.657 (95%CI [ 1.328–1.981]). The risk of positive sputum pathogen cultures was higher in groups with greater DTD-3d. The findings were consistent across different admission periods. Stratified analysis showed that patients without respiratory failure were more affected by DTD-3d, and an interaction effect was observed (*p* < 0.001).

**Conclusion:**

In coastal areas, the diurnal temperature difference three days prior to admission affects the sputum pathogen status in AECOPD patients with respiratory infections.

**Supplementary Information:**

The online version contains supplementary material available at 10.1186/s12890-024-03102-w.

## Introduction

Chronic obstructive pulmonary disease (COPD) has emerged as a globally concerning health issue, imposing a significant economic burden on societies and families [1]. It was estimated that by 2020, the COPD mortality rate would rank third and its disability rate fifth worldwide [2]. Acute exacerbations of COPD (AECOPD) are critical causes of hospital admissions and major contributors to the increased disease burden, worsening pulmonary function, and patient mortality [2, 3].

Several studies have confirmed the association between AECOPD onset and meteorological factors [4–6]. .According to the Global Initiative for Chronic Obstructive Lung Disease (GOLD) guidelines [7], patients with severe AECOPD requiring hospitalization have a higher mortality rate [7], and the majority of AECOPD cases are triggered by respiratory infections [8]. Sputum culture is the most widely used method for detecting respiratory pathogens clinically. The detection of sputum pathogens is a crucial consideration in the clinical management of AECOPD [9]. However, there is limited research discussing the correlation between respiratory pathogen status and meteorological factors in such patients.

A cross-sectional study of adult respiratory infection pathogens in the Xiamen where near Fuzhou identified age and seasonal patterns, with *Rhinovirus* and *Influenza B* peaking in autumn, and elevated Influenza A infections during summer [10]. Contrarily, the infections of the lower respiratory tract were predominantly caused by bacteria or fungi, rather than viruses. A study analyzed bacteria and fungi distribution in lower respiratory tract infections over a year, identifying *Pseudomonas aeruginosa, Candida albicans, Acinetobacter baumannii, Pseudomonas maltophilia*, *Klebsiella pneumoniae* and *Staphylococcus aureus* as the most common pathogens [11]. Although COPD was the most common comorbidity observed in this study, the participant population also included individuals with various underlying conditions such as cardiovascular diseases and malignancies. The study did not allow for an investigation into the correlation between pathogens and local seasonal or atmospheric conditions. Our previous study [11] revealed a decrease in respiratory microbiota diversity and significant changes in community composition among AECOPD patients in our local region.

Therefore, it is valuable for clinical practice to explore the relationship between meteorological factors, microbiota, and AECOPD in COPD patients, particularly in the absence of previous reports from our region. Data were collected from AECOPD patients with concurrent respiratory infections at Fujian Provincial Hospital in Fuzhou, along with local meteorological data. This study focuses on the association between meteorological elements and the detection of sputum pathogens in AECOPD patients with respiratory infections, providing data to assist clinicians in their treatment decisions.

## Methods

### Study population

We conducted a retrospective observational study using data extracted from electronic health records of patients hospitalized with AECOPD at Fujian Provincial Hospital from December 2013 to December 2019. Approval for the study was obtained from the Ethics Committee of Fujian Provincial Hospital (Approval number: K2019-01-003), and all patients provided informed consent for data collection by signing consent forms. The study adhered to the ethical standards of the institution, national research guidelines, the principles of the Helsinki Declaration, and its subsequent amendments.

**Fig. 1:**
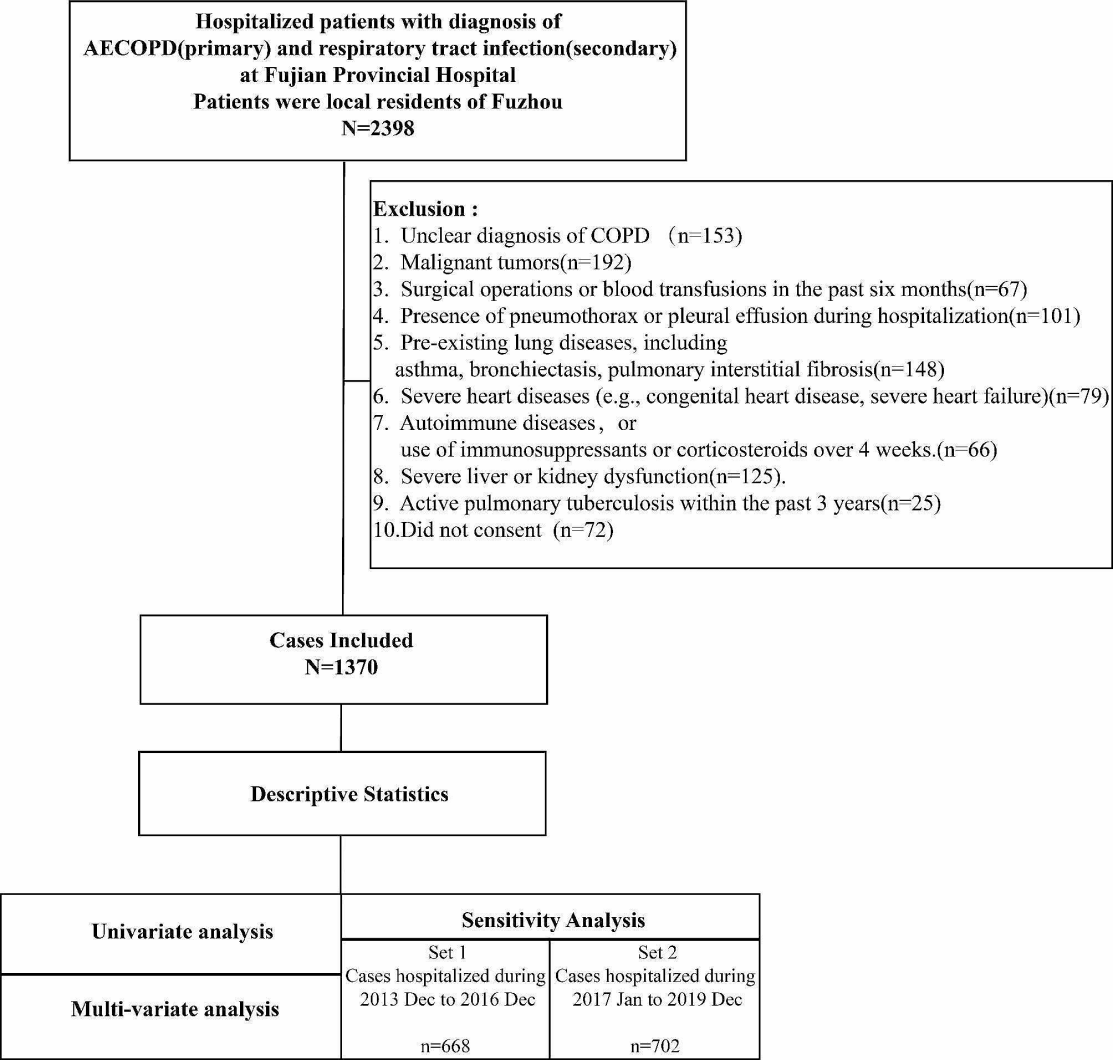
Study flow chart

AECOPD patients hospitalized in Fujian Provincial Hospital from December 2013 to December 2019 were selected and screened according to the flow chart shown in Fig. 1. The inclusion criteria included: (1) Diagnosis of COPD confirmed according to the GOLD guidelines, involving comprehensive review of the patient’s medical history, records, and spirometry data. Specifically, FEV1/FVC ratio of less than 70% after administration of 400 mg Salbutamol indicated COPD. Diagnosis of AECOPD required the presence of at least two primary symptoms (dyspnea, purulent sputum, increased sputum volume) for more than two days, or worsening of these symptoms along with any secondary symptoms (wheezing, sore throat, cough, common cold symptoms); (2) Participants also had diagnoses related to respiratory infections, including pneumonia, bronchitis, pulmonary infections, and lower respiratory tract infections; (3)Participants were local residents in Fuzhou for over three years; (4)The participants had complete clinical record information and consented to data collection. Exclusion criteria included: (1) The diagnosis is not clear; (2) Malignant tumors; (3) Pre-existing lung diseases, including asthma, bronchiectasis, pulmonary interstitial fibrosis; (4) Presence of pneumothorax or pleural effusion during hospitalization; (5)Patients with surgical operations or blood transfusions in the past six months; (6)Severe heart diseases (e.g., myocardial infarct, severe heart failure); (7)Autoimmune diseases, or use of immunosuppressants or corticosteroids over 4 weeks; (8)Active pulmonary tuberculosis occurred within three years prior to admission; (9)Severe liver or kidney dysfunction.

### Measurements

According to the electronic medical order execution records, all measurements were conducted within 24 h of admission. Sputum specimens were collected prior to antibiotic therapy or within 6 h of antibiotic administration. Patients performed mouth rinses and deep coughs to collect sputum. Samples underwent inspection within 2 h, and a smear test ensured qualified specimens, meeting criteria: squamous epithelial cells/white blood cells ratio < 1:2.5. Unqualified samples prompted recollection until meeting standards. Pathogenic bacteria isolation and culture followed National Clinical Examination Operating Procedures. BioMérieux’s VITEK-2 Compact system and automatic analyzer were used, with consumables from BioMerieu (Shanghai). Blood agar, chocolate agar, and MacConkey agar plates were procured from BioMerieu (Shanghai) Biological Products Co., Ltd. According to the standards for sputum culture testing set forth by the Clinical and Laboratory Standards Institute (CLSI) in 2021 (http://em100.edaptivedocs.net/) and the Chinese clinical laboratory standards, a combination of quantitative inoculation and quadrant streaking methods was employed. A positive result was reported if, after 48 h of incubation, the colony area on the agar plate exceeded 5–10% of the total plate area and the preliminary identification indicated common lower respiratory tract pathogens.

Hospitalized patients underwent immediate arterial blood gas analysis. For multiple samples, only the first values were considered. Arterial blood (1 ml) from the radial/femoral artery was collected and analyzed using Radiometer ABL™ 700 (Radiometer Medical Aps, Copenhagen, Denmark). Hypercapnia was defined as PaCO2 ≥ 45 mmHg (6 kPa).

### Meteorological data

Fuzhou is in the southeastern coastal zone of China (N23°33′~28°20′; E115°50′~120°40′), which belongs to the mid-subtropical monsoon climate zone [12]. The meteorological data in Fuzhou during the same period were collected every 5 min. For meteorological element have a lag effect of 3-days based on previous local study [13–15], the analysis included meteorological data from the 3-days preceding the patients’ hospital admission, including the daily average temperature, the diurnal temperature difference(the difference between the maximum and minimum temperatures on the day in meteorology), the atmospheric pressure, the relative humidity, and the wind speed. These data were originated from the Fujian Provincial Meteorological Bureau.

### Statistical analysis

Descriptive statistics were employed to summarize clinical characteristics and meteorological data. Continuous variables are reported as mean ± standard deviation (SD), and categorical variables are depicted as frequencies and percentages. Normality was evaluated using the Shapiro-Wilk test. The student t-test and the Chi-square test were utilized to compare differences across groups. Logistic regression models were developed to analyze the association between key meteorological factors and the detection of pathogens in sputum cultures among patients with AECOPD and respiratory infections, adjusting for age, gender, and respiratory failure. We considered a two-sided p-value of less than 0.05 to indicate statistical significance. All analyses were conducted using IBM SPSS 20.0.

## Results

### Clinical information

A total of 1370 cases were included, with 1105 males and 265 females. Approximately 80% were aged over 70 years old. Respiratory failure was observed in 519 patients. Hospital admissions occurred for 668 cases between 2013 and 2016, and 702 cases from 2017 to 2019. Baseline information for all cases is presented in Table 1.

**Table 1:** Clinical baseline of hospitalized AECOPD accompany airway infection

	Total	Admission	Admission	*p*-chi test
	(2013–2019)	(2013–2016)	(2017–2019)	
Total number of cases	1370	668	702	
Gender				0.125
Male, n (%)	1105 ( 80.66 )	550 ( 82.34 )	555 ( 79.06 )	
Female, n (%)	265 ( 19.34 )	118 ( 17.66 )	147 ( 20.94 )	
Age ( years)				0.786
<70, n (%)	475 ( 34.67 )	234 ( 35.03 )	241 ( 34.33 )	
≥ 70, n (%)	895 ( 65.33 )	434 ( 64.97 )	461 ( 65.67 )	
Smoking history				0.165
Never, n (%)	347 ( 25.32 )	161 ( 24.10 )	186 ( 26.49 )	
Cessation, n (%)	841 ( 61.39 )	407 ( 60.93 )	434 ( 61.82 )	
Smoking, n (%)	182 ( 13.28 )	100 ( 14.97 )	82 ( 11.69 )	
Respiratory failure				0.602
Yes, n (%)	519 ( 37.88 )	249 ( 37.28 )	270 ( 38.46 )	
No, n (%)	851 ( 62.12 )	419 ( 62.72 )	432 ( 61.54 )	
Sputum Culture Pathogens				0.439
Positive, n (%)	578 ( 42.19 )	279 ( 41.76 )	299 ( 42.59 )	
Negative, n (%)	792 ( 57.81 )	389 ( 58.24 )	403 ( 57.41 )	

**Fig. 2:**
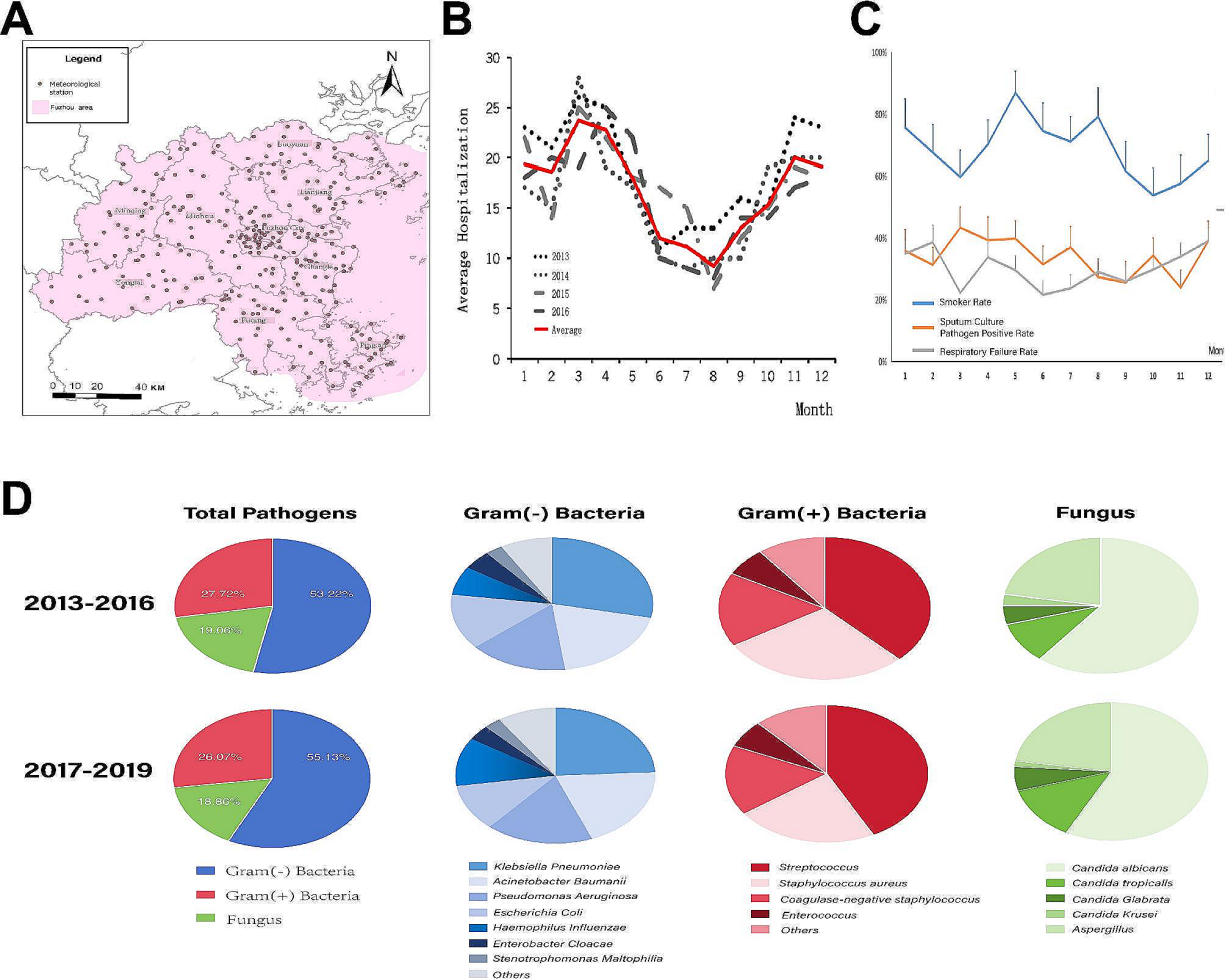
Overview of Hospital Admissions for AECOPD and Pathogen distribution in sputum culture in Fuzhou. (**A**) The distribution map of regional meteorological stations. (**B**) Distribution of the number of monthly AECOPD hospitalizations. (**C**) Distribution on the positive rate of sputum culture and respiratory failure rate in hospitalized AECOPD. (**D**) Pathogen distribution in sputum culture of hospitalized AECOPD patients with airway infection.

Situated in the coastal zone (N23°33′~28°20′; E115°50′~120°40′), Fuzhou has a subtropical monsoon climate. The distribution of meteorological monitoring stations is shown in Fig. 2A. Hospitalizations for AECOPD accompany with airway infection peaked from March to May, with a secondary increase from November to January the following year. A decline in hospitalizations was noted from June to September (Fig. 2B). Clinical data showed an increased positive rate for sputum culture from March to May and a higher incidence of respiratory failure in these AECOPD patients from November to February the following year (Fig. 2C).

Of all the cases, 578 (42.19%) had a positive sputum culture report indicating pathogen growth. Among these, the period from 2013 to 2016 saw 279 positive cases, with Gram-negative bacteria being the most detected (53.22%), followed by Gram-positive bacteria (27.72%), and fungi detected in 19.05% of cases. The detection pattern was similar in the period from 2017 to 2019. In the two groups studied, the most detected Gram-negative bacteria were *Klebsiella pneumoniae, Pseudomonas aeruginosa, Acinetobacter baumannii, Escherichia coli*, and *Haemophilus influenzae.* The most frequently identified Gram-positive bacteria were Streptococcus pneumoniae and Staphylococcus aureus, while the most common fungi detected were Candida albicans and Aspergillus. Figure 2D shows the distribution of detected sputum pathogens.

### Univariate analysis revealed significant differences among patients with different sputum pathogen culture results in DTD and ARH in the three days before hospital admission

After grouping according to sputum pathogen culture results, a t-test analysis of daily meteorological element variations three days before hospital admission (see Table 1) indicated that patients with positive sputum culture pathogens experienced significantly larger Diurnal Temperature Difference (DTD) compared to those with negative cultures. Following stratification by hospitalization duration, there was a consistency in the population with a significantly larger DTD in three days prior to admission (DTD-3d) among patients in the sputum culture positive group. Compared to patients with negative sputum culture results, those with positive sputum culture had higher Average Relative Humidity (ARH) in three days prior to admission, indicating a significant difference. Stratifying by hospitalization duration, patients in the sputum culture positive group exhibited a slightly higher ARH in both groups, which was statistically significant. No significant difference in the daily mean temperature, daily mean wind speed, and daily mean atmospheric pressure three days prior to admission among patients with different sputum culture results were observed in this study.

**Table 2:** Univariate analysis of sputum pathogen culture in AECOPD patients with concurrent respiratory infections and meteorological factors

Meteorological factor (3days before admission)	Total_Sputum Culture			2013–2016_Sputum Culture			2017–2019_Sputum Culture		
	Positive (*n* = 597)	Negative (*n* = 773)	*p* ttest	Positive (*n* = 284)	Negitive (*n* = 384)	*p* ttest	Positive (*n* = 313)	Negitive (*n* = 389)	*p* ttest
Average atmospheric pressure (AAP), hPa	1003.74 ± 9.99	1010.02 ± 10.63	0.552	999.98 ± 11.02	1009.98 ± 9.94	0.476	1006.56 ± 10.17	1010.81 ± 10.04	0.464
Average temperature (AT), ℃	13.15 ± 10.46	15.95 ± 11.39	0.332	12.88 ± 11.17	15.76 ± 11.73	0.414	13.43 ± 9.86	16.56 ± 10.17	0.443
Diurnal temperature difference (DTD), ℃	10.76 ± 4.73	7.07 ± 2.81	0.019	9.29 ± 4.75	6.92 ± 2.24	0.027	11.82 ± 4.51	8.15 ± 3.39	0.025
Average relative humidity (ARH), %	77.43 ± 19.86	68.21 ± 17.17	0.038	75.17 ± 20.68	65.92 ± 17.17	0.051	78.95 ± 19.59	70.41 ± 19.74	0.046
Average wind speed (AWS), m/s	3.1 ± 0.3	3.0 ± 0.3	0.279	3.0 ± 0.4	2.8 ± 0.3	0.340	3.1 ± 0.3	3.2 ± 0.4	0.288

### Multi-variable logistic regression analysis indicates (DTD-3d) is associated with the sputum culture status of hospitalized patients with concomitant airway infections and AECOPD

In the multivariable logistic regression analysis model included the diurnal temperature range three days prior to admission (based on quartile division), average relative humidity, age, gender, and respiratory failure (Table 3). DTD-3d ≥ 10℃(OR 1.65, 95%CI [1.33–1.98]), ARH-3d > 75% (OR 1.47, 95%CI [1.05–2.00]), and concomitant respiratory failure (OR 2.06, 95%CI [1.15–2.98]) were independent risk factors for positive sputum pathogen culture in hospitalized patients with concomitant airway infections and AECOPD. When the DTD-3d was considered in quartiles, compared to those in the smallest quartile (0–6 ℃), these patients with a DTD-3d of 6–10℃ (OR 1.45, 95%CI [0.91–2.02]), 11–15℃ (OR 1.76, 95%CI [1.03–2.56]) and > 15℃ (OR 2.43, 95%CI [1.26–3.61]) were more likely to have positive sputum pathogen cultures. Repeated analysis in populations admitted at different times showed consistent trends.

**Table 3:** Multivariable logistic regression analysis of diurnal temperature difference and sputum pathogen detection in hospitalized AECOPD patients with concurrent respiratory infections

		2013–2016		2017–2019		Total	
		OR 95%CI	*p*	OR 95%CI	*p*	OR 95%CI	*p*
Diurnal Temperature Difference							
0–6°C							
6–10°C		1.45 [0.91–2.02]	0.046	1.49 [0.99–1.93]	0.048	1.47 [0.96–2.18]	0.038
11–15°C		1.76 [1.03–2.56]	0.019	1.71 [1.04–2.40]	0.023	1.73 [1.10–2.37]	0.012
>15°C		2.43 [1.26–3.61]	< 0.001	2.40 [1.24–3.55]	< 0.001	2.42 [1.56–3.29]	< 0.001
Average Relative Humidity							
<75%							
≥ 75%		1.47 [1.05–2.00]	0.041	1.42 [1.07–1.89]	0.048	1.46 [1.10–1.85]	0.033
Age							
≤ 70							
>70		1.31 [0.97–1.65]	0.153	1.25 [0.99–1.57]	0.127	1.27 [0.98–1.54]	0.220
Gender							
Female							
Male		1.60 [0.81–2.37]	0.311	1.54 [1.03–2.07]	0.278	1.57 [1.00–2.03]	0.189
Respiratory Failure							
No							
Type I		2.06 [1.15–2.98]	< 0.001	1.83 [1.22–2.75]	< 0.001	2.03 [1.20–2.89]	< 0.001

### Interaction between DTD-3d and concurrent ARH

**Fig. 3:**
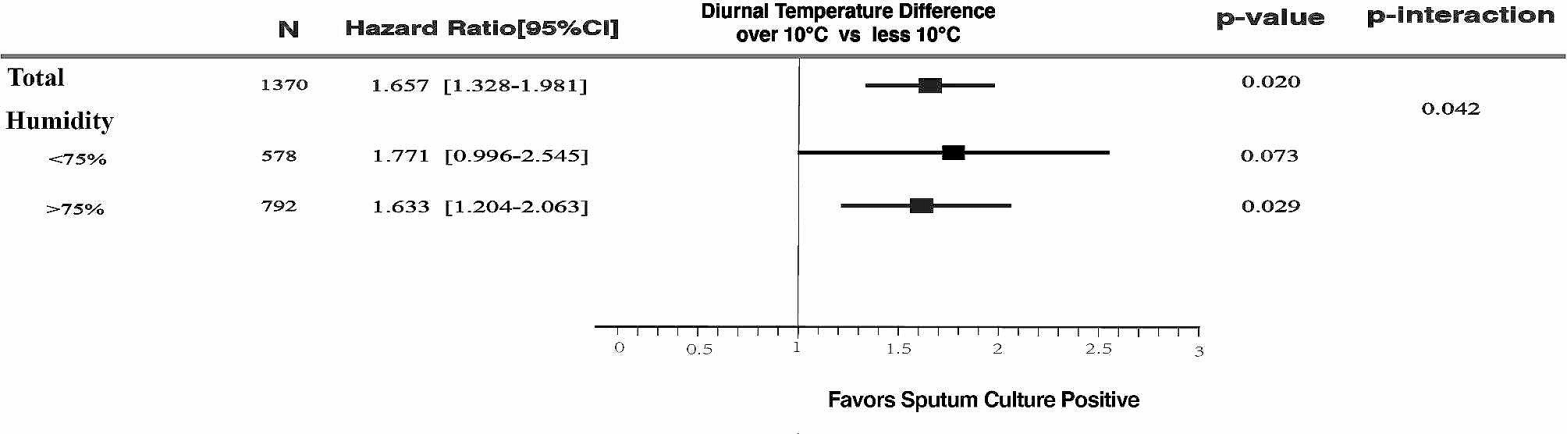
Stratified Analysis of Diurnal Temperature Range and Humidity on Sputum Pathogen Detection in Hospitalized AECOPD Patients with Concurrent Respiratory Infections

Figure 3 indicated a potential interaction between DTD-3d and concurrent ARH on the sputum culture status of hospitalized patients with concomitant airway infections and AECOPD (p-interaction = 0.042). In conditions of AHR > 75%, patients had a greater risk of positive sputum pathogen culture when the DTD-3d > 10℃ (OR 1.633, 95% CI[1.204–2.063]), significantly. In case of AHR<75%, the increased risk of positive sputum pathogen culture in patients with a DTD-3d > 10℃ did not show statistical significance.

Table 4 showed the additive interaction between humidity and DTD-3d on the positive sputum culture results in hospitalized COPD patients. In the general population of this region, the combined exposure to humidity and a DTD-3d(>10 °C) had a synergistic additive interaction on the increased risk of positive sputum cultures (RERI 0.08, 95%CI [0.01, 0.14]). In these groups, approximately 9% of the composite risk for positive sputum cultures were attributable to the interaction between humidity and substantial DTD-3d (AP [95%CI] as [0.03, 0.14], [0.01, 0.20] and [0.02, 0.15] respectively). The results suggested that in this region, the combined exposure to dryness and DTD-3d (>10 °C) might have an antagonistic interaction effect on the risk of positive sputum cultures (-0.07 [-0.14, -0.01]).

**Table 4:** Interaction between different humidity conditioning and DTD-3d

Groups	n	RERI [95%CI]	AP [95%CI]	SI [95CI%]
**Additive Interaction of Wet-DTD(>10 °C) on Sputum Pathogen Culture**				
Total	1370	0.08 [0.01,0.14]	0.06[0.01,0.11]	1.34[1.03,1.75]
Gender				
Male	1105	0.10[0.03,0.19]	0.09[0.03,0.14]	1.71[1.18,2.65]
Female	265	0.02[-0.07,0.13]	0.01[-0.04,0.08]	1.11[0.77,1.49]
Age (years)				
<70	475	0.05[-0.03,0.15]	0.03[-0.05,0.12]	1.25[0.72,2.10]
≥70	895	0.07[0,01,0.18]	0.09[0.01,0.20]	1.40[1.06,2.23]
Smoking history				
Never	347	-0.01[-0.10,0.11]	0.06[-0.07,0.20]	0.99[0.75,1.34]
Cessation	841	0.04[-0.02,0.12]	0.05[-0.03,0.13]	1.20[0.89,1.60]
Smoking	182	0.11[0.03,0.21]	0.09[0.02,0.15]	1.42[1.09,2.02]
**Additive Interaction of Dry-DTD(>10 °C) on Sputum Pathogen Culture**				
Total	1370	-0.07 [-0.14,-0.01]	-0.06[-0.12,-0.01]	0.65[0.41,0.88]
Gender				
Male	1105	-0.11[-0.18,-0.04]	-0.11[-0.17,0.04]	0.43[0.28,0.61]
Female	265	-0.03[-1.00,0.08]	-0.02[-0.08,0.05]	0.83[0.47,1.39]
Age (years)				
<70	475	-0.04[-0.14,0.06]	-0.02[-0.10,0.05]	0.62[0.27,1.64]
≥70	895	-0.09[-0.20,-0.01]	-0.08 [-0.16,-0.01]	0.56[0.31,0.87]
Smoking history				
Never	347	-0.11[-0.34,0.07]	-0.12[-0.35,0.09]	0.31[0.02,1.51]
Cessation	841	-0.07[-0.21,0.05]	-0.04[-0.14,0.07]	0.67[0.41,1.04]
Smoking	182	-0.08[-0.19,-0.01]	-0.09[-0.19,-0.01]	0.54[0.26,0.89]

## Discussion

### Exploring research results

We investigated the link between meteorological factors and the sputum culture status of hospitalized patients with concomitant airway infections and AECOPD, as the results of sputum culture play a significant role in guiding clinical treatment for these patients, and AECOPD is often associated with meteorological changes. Firstly, our descriptive statistical analysis of 6-year data from our center revealed that AECOPD patients with airway infections exhibited seasonal distribution characteristics, with over 40% showing pathogen growth in sputum cultures, predominantly Gram-negative bacteria. Subsequently, based on the lag time for meteorological factor effects documented in the literature, our univariate analysis revealed differences in DTD-3d and AHR-3d between patients with different sputum culture statuses. Further, multivariate logistic regression analysis was conducted to explore whether daily temperature range is an independent factor for the occurrence of positive sputum cultures in these patients, followed by a sensitivity analysis. Meanwhile, ARH-3d and DTD-3d might had the additive interaction on the positive sputum culture results in hospitalized COPD patients. Additionally, stratified analysis identified that older and male AECOPD patients might be a relatively susceptible group to pathogen growth in sputum with increased DTD-3d. Our results highlighted that an increased DTD-3d may be a contributing factor to the presence of positive sputum pathogens in hospitalized AECOPD patients with airway infections, particularly in subtropical coastal areas.

Respiratory infections are the most common accelerators of AECOPD. Unlike most studies, our research included cases diagnosed with both AECOPD and respiratory infections, employing the clinical widely used sputum test technique. We report an increase in the positivity rate of sputum bacterial cultures among hospitalized AECOPD patients from March to May, correlating with humidity and temperature. The sputum cultures exhibited a high positive rate, predominantly with Gram-negative bacteria consistent with some previous reports [16–18]. A pediatric study observed significant seasonal variations in the detection of respiratory bacterial pathogens, suggesting a possible link between the Gram-negative bacteria and environmental temperatures [19]. A multicenter study in the United States reported that both geographical latit, a higher prevalence of *Methicillin-resistant Staphylococcus Aureus* and a lower prevalence of *Vancomycin Resistant Enterococci* were observed as one moves southward in latitude [20]. Our previous study [16] has shown a notable decrease in respiratory microbial diversity and significant shifts in AECOPD patients, with a significant elevated abundance of *Haemophilus, Moraxella, Klebsiella, and Pseudomonas* species. Similarly, a cohort study [21] suggests that increased winter AECOPD incidence potentially due to a rise in pathogen presence, including *Haemophilus influenzae* infections and viral infections. However, some studies were limited to stable phase or outpatient AECOPD patients, whereas our study focuses on hospitalized patients, among whom more virulent bacteria, including key pathogens like *Pseudomonas aeruginosa, Acinetobacter baumannii, Klebsiella pneumoniae and Escherichia* coli etc. In summary, we have established a foundation for understanding the onset of AECOPD from the perspective of infection influenced by meteorological factors [22].

The fluctuation in airway microbial loads, predominantly Gram-negative bacteria, is closely linked to seasonal factors affecting the airway’s self-cleaning capacity. Mely low temperatures is associated with more severe AECOPD [23, 24]. However, our model, conducted in relatively warm regions, suggests that temperature differences, rather than average temperatures, play a crucial role in the incidence of AECOPD. This finding is corroborated by multicenter studies, which confirm the impact of temperature differences on COPD hospitalizations or mortality [23, 25]. This study focuses primarily on sputum culture results which may affect antibiotic treatment decisions. Multifactorial analysis found that greater temperature variation appears to have a clearer impact on the growth of sputum pathogens in AECOPD hospitalized patients. Potential mechanisms considered include fluctuations in respiratory epithelial temperature, which can affect the respiratory system’s host defense functions, nasal responses, and the clearance of airway mucosal cilia [26, 27], and may enhance the transmission of viruses and bacteria, leading to an exacerbation of respiratory diseases [28].

### Clinical implications

The impact of meteorological factors on respiratory diseases is often complex and interactive [24]. While many epidemiological studies have focused on single meteorological variables such as humidity or temperature and their impact on health, individuals were exposed to both. Therefore, we explored the stratified and interactive analysis of temperature and humidity in the detection of respiratory bacteria in hospitalized COPD patients, drawing valuable insights from real-world data, which are currently rare. The results suggest that under high humidity conditions, DTD-3d have a significant impact on the sputum culture status of AECOPD patients. A small cohort study conducted in Shanghai observed that low temperatures are a risk factor for COPD, and high humidity increases the risk of COPD induced by low temperatures [22]. Similarly, a retrospective study on disease burden reported a positive correlation between humidity and COPD mortality [29]. A cross-sectional study in Canada found that the number of AECOPD significantly increased under hot and humid conditions [30]. In our analysis, temperature changes and humidity also had an interactive effect on the sputum culture status (p-interaction < 0.05). Considering that biological interactions are evaluated on an additive scale, we adopted an additive interaction model for analysis the combined effects of meteorological factors. We found that greater temperature variations combined with humid conditions have a synergistic interactive effect on increasing the risk of detecting respiratory pathogens in the COPD population, with a potential antagonistic interaction in dry conditions with large temperature variations. These could be explained by the fact that cold air can cause bronchoconstriction, leading to difficulty breathing, increased symptom exacerbations. Additionally, cold weather can also increase the risk of respiratory infections, which can further worsen symptoms in individuals with COPD [5]. In humid environments, the presence and survival of allergens and pathogens in the air are increased, which are then inhaled into the respiratory tract by patients, exacerbating respiratory symptoms in individuals with COPD and asthma, and triggering inflammatory responses [31, 32]. Furthermore, air pollutants also played a mediating role in the short-term association between humidity, temperature, and clinical outcomes of COPD [33]. However, extensive clinical epidemiological research and experimental animal studies are still needed to explore the biological mechanisms of the effects of combined exposure to temperature variation and humidity on COPD patients.

In the stratified analysis, we observed that the effects of combined exposure to low temperatures and humidity on the detection of sputum bacteria in hospitalized COPD patients vary by age. Specifically, the risk of sputum bacteria detection is higher in the elderly population (aged over 70 years) compared to relatively younger individuals. This may be due to the poorer physical condition of the elderly, their higher prevalence of underlying diseases, and their diminished capacity for thermoregulation and maintaining homeostasis, which increases respiratory risks [34, 35]. Furthermore, gender differences in response to various combined exposure scenarios suggest physiological differences between genders, varying lifestyle and dietary habits, and unequal exposure durations [36, 37]. Additionally, the sputum culture results from patients who persisted in smoking exhibit notable susceptibility to temperature variations and humidity. Nevertheless, the limited sample size hinders the establishment of definitive conclusions regarding the influence of smoking. Further studies are needed to explore the biological mechanisms involved. Our findings also indicate that individuals with different smoking statuses may respond differently to these combined exposure events. Specifically, those who are currently smoking showed more pronounced effects under combined low temperature and humidity exposure. Although it is hypothesized that smoking may impair airway regulatory ability, increasing susceptibility to environmental factors, the biological evidence is still insufficient. Future prospective studies are hoped to further elucidate these results.

### Strengths and limitations

The strength of this study lies in its being one of the few investigations into the relationship between respiratory infection pathogens and meteorological conditions. Our criteria facilitated the selection of hospitalized patients with AECOPD combined with respiratory infections. Reliable clinical data aided in exploring the impact of meteorological changes on the pathogens in patients’ sputum, which is a critical consideration for clinicians in treatment. Various analyses helped us ascertain the relevance of potential confounding factors. Moreover, our research across populations admitted at different times confirmed the stability of our conclusions.

Our study still has limitations. As a single-center retrospective study, our model requires prospective validation in independent COPD patients from other healthcare systems. Currently, we have only measured meteorological data at a single time point (three days prior to admission) and demonstrated a correlation. Further investigation into temperature differences over various time lags could explore their impact on AECOPD hospitalizations. While sputum culture is widely used clinically, emerging technologies like next-generation sequencing may help explore changes in the overall respiratory microbiome of AECOPD hospitalized patients under the influence of temperature variations, rather than just specific strains.

## Conclusion

Our study highlights an increased risk of positive sputum pathogen culture in hospitalized patients with AECOPD and respiratory infections in the coastal regions of southern China experiencing greater diurnal temperature variations three days prior to admission. Information regarding temperature fluctuations can be readily accessed, and these findings provide clinicians with epidemiological and microbiological data for considering antibiotic therapy decisions in this context.

### Electronic supplementary material

Below is the link to the electronic supplementary material.

Supplementary Material 1

## Data Availability

The data utilized in this study were collected and analyzed by the researchers from the enrolled patients. Meteorological and environmental data were supplied by the unit affiliated with Chunhui Wang and Wei Zheng, at the Fujian Provincial Meteorological Bureau. The datasets used and/or analyzed during the current study available from the corresponding author on reasonable request.

## Abbreviations

COPD: Chronic Obstructive Pulmonary Disease
AECOPD: Acute Exacerbation of COPD
GOLD: Global initiative for chronic obstructive lung disease
DTD: Diurnal Temperature Differences
AHR: Average Relative Humidity
FEV1: Forced expiratory volume in 1 s
FVC: Forced vital capacity
