# Supplementary material for: Impact of diurnal temperature variations on sputum bacterial detection in hospitalized patients with acute COPD exacerbation: a retrospective study from Fuzhou, China
Source: BMC Pulm Med. 2024 Jun 22;24:291. doi: 10.1186/s12890-024-03102-w (PMC11193170; doi:10.1186/s12890-024-03102-w)
Supplement: Supplementary file 1 — Supplementary Material 1 [file 12890_2024_3102_MOESM1_ESM.docx]

**Supplement Data**

**Table S1.Meteorological data in Fuzhou (2013 ~ 2016)**

| **Mon** | **Temperature Difference**  **(℃)** | **Average Temperature**  **(℃)** | **Atmospheric Pressure**  (kPa) | **Relative Humidity**  **(%)** | **Wind Velocity**  **(m/s)** | **Sunshine Time**  (h) |
| --- | --- | --- | --- | --- | --- | --- |
| 1 | 7.3±2.4 | 11.2±3.6 | 101.4±0.27 | 71.1±15.4 | 2.1±0.1 | 5.3±3.2 |
| 2 | 7.3±4.3 | 11.3±3.4 | 101.5±0.31 | 71.8±13.9 | 2±0.2 | 2.4±2.8 |
| 3 | 8.2±3.0 | 13.7±3.4 | 101.0±0.45 | 79.6±7.7 | 2±0.2 | 3.1±3.8 |
| 4 | 8.3±3.1 | 19.7±2.7 | 100.3±0.31 | 84.2±10.6 | 2.2±0.1 | 4±3.3 |
| 5 | 7.0±2.8 | 24.1±2.8 | 100.1±0.26 | 80.4±9.4 | 2±0.3 | 5.1±4.4 |
| 6 | 6.9±2.3 | 27.8±2.4 | 99.81±0.4 | 78.8±7.8 | 2.3±0.2 | 5.8±3.6 |
| 7 | 6.7±2.3 | 29.8±1.4 | 99.7±0.26 | 73.6±7.8 | 2.6±0.1 | 7.7±4.2 |
| 8 | 6.4±1.9 | 30.1±1.5 | 99.5±0.22 | 63.4±6.6 | 2.7±0.1 | 6.1±4.2 |
| 9 | 6.6±2.0 | 26.7±1.3 | 99.9±0.46 | 69.3±6.1 | 2.1±0.2 | 5.2±3.7 |
| 10 | 5.5±2.6 | 24.7±2.7 | 100.4±0.43 | 63.9±7.9 | 2.2±0.3 | 5.4±3.3 |
| 11 | 5.8±2.7 | 18.7±3.4 | 101.0±0.37 | 77.3±10.5 | 2±0.1 | 2.3±3.4 |
| 12 | 6.8±2.9 | 12.3±3.2 | 101.3±0.39 | 69.5±10 | 2.1±0.1 | 3.8±3.6 |

Note: data were expressed as mean ± standard deviation

| **Table S2 Correlation analysis of air pollutants in Fuzhou and clinical data of AECOPD from 2013-2019** | | | | | | |
| --- | --- | --- | --- | --- | --- | --- |
|  |  |  |  |  |  |  |
| **Pollutants (μg/m^3^)** | **Number of AECOPD hospitalization** | | | **Detection rate of pathogenic bacteria in sputum of AECOPD patients** | | |
|  | **OR** | **95%CI** | ***p*** | **OR** | **95% CI** | ***p*** |
| PM_2.5_ (μg/m^3^) | 1.12 | 1.07-1.17 | <0.01 | 1.01 | 1.97-1.05 | 0.155 |
| PM_10_ (μg/m^3^) | 1.08 | 1.05-1.11 | <0.01 | 1.06 | 1.03-1.09 | <0.05 |
| NO_2_ (μg/m^3^) | 1.01 | 1.00-1.02 | <0.05 | 1.01 | 0.99-1.03 | 0.189 |
| SO_2_ (μg/m3) | 1 | 0.98-1.02 | 0.378 | 1.01 | 0.97-1.02 | 0.229 |
| CO (mg/m3) | 0.98 | 0.93-1.01 | 0.211 | 0.97 | 0.94-1.00 | 0.252 |
